# A New Classification of Endodontic-Periodontal Lesions

**DOI:** 10.1155/2014/919173

**Published:** 2014-04-14

**Authors:** Khalid S. Al-Fouzan

**Affiliations:** College of Dentistry, King Saud Bin Abdulaziz University, King Abdulaziz Medical City No. 1243, P.O. Box 22490, Riyadh 11426, Saudi Arabia

## Abstract

The interrelationship between periodontal and endodontic disease has always aroused confusion, queries, and controversy. Differentiating between a periodontal and an endodontic problem can be difficult. A symptomatic tooth may have pain of periodontal and/or pulpal origin. The nature of that pain is often the first clue in determining the etiology of such a problem. Radiographic and clinical evaluation can help clarify the nature of the problem. In some cases, the influence of pulpal pathology may cause the periodontal involvement and vice versa. The simultaneous existence of pulpal problems and inflammatory periodontal disease can complicate diagnosis and treatment planning. An endo-perio lesion can have a varied pathogenesis which ranges from simple to relatively complex one. The differential diagnosis of endodontic and periodontal diseases can sometimes be difficult, but it is of vital importance to make a correct diagnosis for providing the appropriate treatment. This paper aims to discuss a modified clinical classification to be considered for accurately diagnosing and treating endo-perio lesion.

## 1. Introduction

The periodontal-endodontic lesions have been characterized by the involvement of the pulp and periodontal disease in the same tooth. This makes it difficult to diagnose because a single lesion may present signs of both endodontic and periodontal involvement. There is a general agreement today that the vast majority of pulpal and periodontal lesions are the result of bacterial infection. This suggests that one disease may be the result or cause of the other or even originated from two different and independent processes which are associated with their advancement [1]. Diagnosis is complicated by the fact that these diseases are too frequently viewed as independent entities. However, it is critical to recognize the interrelationship for successful management of these lesions. The pathways for the spread of bacteria between pulpal and periodontal tissues are still a subject of controversy [2–6].

The apical foramen is the main access route between the pulp and the periodontium, with the participation of all root canal system: accessory, lateral, and secondary canals, as well as the dentinal tubules through which the bacteria and its products contaminate the medium [7, 8]. It is known that the main cause of the periodontal lesions is the presence of the bacterial plaque, formed by aerobic and anaerobic microorganisms [9–12]. Pulp exposures, periodontitis, and caries lesions are of significant importance in the development of periodontal-endodontic lesions. If the lesions are not well treated and the canals are not disinfected and sealed completely, they will house bacterial necrotic rests, which account for the progression of the lesion or even for the endodontic reinfection [13–15]. Another form of the interrelationship is because of the iatrogenic perforations due to either rotary instruments or improper handling of the endodontic instruments [16].

Vertical root fractures and cracks may serve as a “bridge” for pulp contamination. If the periodontium had a previous inflammation, it may lead to dissemination of the inflammation which can result in pulp necrosis [17].

Several authors, through their studies, diverge on the contamination routes. Rubach and Mitchell [18] suggested that the periodontal disease may affect the pulp health when the accessory canal exposure occurs, allowing the periodontopathogenic bacteria to cause inflammatory reactions followed by pulp necrosis.

Lindhe [19] also reported that bacterial infiltrates of the inflammatory process may reach the pulp when there is accessory canal exposure, through apical foramens and canaliculi of the furcation area. Adriaens et al. [8] demonstrated that bacteria coming from the periodontal pockets have the capacity of reaching the root canals towards the pulp, suggesting that the dentinal tubules may serve as a reservoir for these microorganisms and that a recolonization of the treated root surface may occur.

It is highlighted that the root planning and scaling may result in the rupture of the vessels and destruction of the neurovascular bundle in the lateral canals, provoking a reduction of the blood supply and consequently leading to pulp alterations.

Knowledge of these disease processes is essential in coming to the correct diagnosis. This is achieved by careful history taking, examination, and performing special tests.

This paper is an attempt to provide a rational classification to the endo-perio question in order to scientifically diagnose and treat these lesions with predictable success.

The periodontal-endodontic lesions have received several classifications, among which is the classification of Simon et al. [20] separating lesions involving both periodontal and pulpal tissues into the following groups:

- primary endodontic lesions,
- primary endodontic lesions with secondary periodontal involvement,
- primary periodontal lesions,
- primary periodontal lesions with secondary endodontic involvement,
- true combined lesions.

From the point of view of treating these cases efficaciously, another clinical classification was provided by Torabinejad and Trope in 1996 [21], based on the origin of the periodontal pocket:

- endodontic origin,
- periodontal origin,
- combined endo-perio lesion,
- separate endodontic and periodontal lesions,
- lesions with communication,
- lesions with no communication.

Another classification was recommended by the world workshop for classification of periodontal diseases (1999) [22], Periodontitis Associated with Endodontic Disease:

- endodontic-periodontal lesion,
- periodontal-endodontic lesion,
- combined lesion.

Based on these classifications, the most widely used classification of endodontic-periodontal lesions is the one that has been classified by Simon et al. [20], according to the primary cause of disease. One of the main classification items was primary endodontic disease, which we believe should be modified, since it has no periodontal relationship. A new endodontic-periodontal interrelationship classification, based on the primary disease with its secondary effect, is suggested as follows:

1. retrograde periodontal disease:
  - primary endodontic lesion with drainage through the periodontal ligament,
  - primary endodontic lesion with secondary periodontal involvement;
2. primary periodontal lesion;
3. primary periodontal lesion with secondary endodontic involvement;
4. combined endodontic-periodontal lesion;
5. iatrogenic periodontal lesions.

*(1) Retrograde Periodontal Disease.* It could be of two subcategories.

*(a) Primary Endodontic Lesion with Drainage through the Periodontal Ligament*. A deep narrow probing defect is noted on just one aspect of the tooth root. Acute exacerbation of a chronic apical lesion on a tooth with a necrotic pulp may drain coronally through the periodontal ligament into the gingival sulcus. This condition may mimic, clinically, the presence of a periodontal abscess. In reality, it is a sinus tract from pulpal origin that opens through the periodontal ligament area. For diagnostic purposes, it is imperative for the clinician to insert a gutta-percha cone into the sinus tract and to take one or more radiographs to determine the origin of the lesion. When the pocket is probed, it is narrow and lacks width. Primary endodontic diseases usually heal following root canal treatment.

*(b) Primary Endodontic Lesion with Secondary Periodontal Involvement.* There is a more extensive periodontal pocket which has occurred as a result of the drainage from noxious agents present in an infected root canal system. Long-term existence of the defect has resulted in deposits of plaque and calculus in the pocket with subsequent advancement of the periodontal disease.

The integrity of the periodontium will be reestablished if root canal treatment is done properly. If a draining sinus tract through the periodontal ligament is present before root canal treatment, resolution of the probing defect is expected.

*(2) Primary Periodontal Lesion.* The periodontal disease has gradually spread along the root surface towards the apex. The pulp may remain vital but may show some degenerative changes over time. In such cases, it is advisable to treat the periodontal tissues only.

*(3) Primary Periodontal Lesion with Secondary Endodontic Involvement*. Progression of the periodontal disease and the pocket leads to pulpal involvement via either a lateral canal foramen or the main apical foramen. The pulp subsequently becomes necrotic and infected. In such cases, it is advisable to treat both tissues [23].

*(4) Combined Endodontic-Periodontal Lesion*. The tooth has a pulpless, infected root canal system and a coexisting periodontal defect. A simpler classification would be to define any situation with both endodontic and periodontal diseases as being a “combined endodontic-periodontal lesion.” An attempt should be made to identify the primary cause of a combined lesion but this may not always be possible. In such cases, it is not essential to determine which disease entity occurred first as the treatment will involve both endodontic and periodontal management. If only one of the problems was treated, then it would be expected that the lesion would not heal adequately. It is generally advisable to treat both tissues concurrently in order to create the most favorable environment for healing.

*(5) Iatrogenic Periodontal Lesions.* Lesions produced as a result of treatment modalities include the following. 

*(A) Root Perforations*. Iatrogenic root canal perforations: they are serious complications during dental treatment and have a rather poor prognosis [24]. Perforations may be produced by powered rotary instruments during the attempt to gain access to the pulp or during preparation for a post. Improper manipulation of endodontic instruments can also lead to a perforation of the root. When root perforation occurs, communications between the root canal system and either periradicular tissues or the oral cavity may often reduce the prognosis of treatment. At the site of perforation, an inflammatory reaction in periodontal ligament occurs and leads to the formation of a lesion which can progress as a conventional primary endodontic lesion.

*(B) Coronal Leakage.* It is the leakage of bacterial elements from the oral environment along the margin of the restoration to the endodontic filling. Studies have indicated that this factor may be an important cause of endodontic treatment failure [25–27]. Root canals may become recontaminated by microorganisms due to delay in placement of a coronal restoration and fracture of the coronal restoration and/or the tooth. Madison and Wilcox [13] found that exposure of root canals to the oral environment leads to coronal leakage, and in some cases along the entire length of the root canal. Ray and Trope [14] reported that defective restorations and adequate root canal fillings had a higher incidence of failures than teeth with inadequate root canal fillings and adequate restorations.

*(C) Dental Injuries or Trauma.* They may take on many shapes but generally can be classified as enamel fractures, crown fractures without pulp involvement, crown fractures with pulp involvement, crown-root fracture, root fracture, luxation, and avulsion [28]. Treatment of traumatic dental injuries varies depending on the type of injury and it will determine pulpal and periodontal ligament healing prognosis [17, 29–33]. The most common cause of vertical root fracture in endodontically treated teeth is the excessive force used during lateral condensation of gutta-percha. Mild pain or discomfort and swelling are the major clinical symptoms, and solitary pocket around one aspect of the suspected tooth is the major clinical sign.

*(D) Chemicals Used in Dentistry*. They have the potential to cause root resorption. Clinical reports [34–36] have shown that intracoronal bleaching with highly concentrated oxidizing agents, such as 30–35% hydrogen peroxide, can induce root resorption. The irritating chemical may diffuse through the dentinal tubules, and when combined with heat, they are likely to cause necrosis of the cementum, inflammation of the periodontal ligament, and subsequently root resorption [36, 37]. Replacement resorption or ankylosis occurs following extensive necrosis of the periodontal ligament with formation of bone onto a denuded area of the root surface. This condition is most often seen as a complication of luxation injuries, especially in avulsed teeth that have been out of their sockets in dry conditions for several hours. The potential for replacement resorption was also associated with periodontal wound healing. Granulation tissue derived from bone or gingival connective tissue may induce root resorption and ankylosis [17, 31].

*(E) Vertical Root Fractures.* The artificial pathways between periodontal and pulpal tissues are vertical root fractures. Vertical root fractures are caused by trauma and have been reported to occur in both vital and nonvital teeth. In vital teeth, vertical fractures can be continuations of coronal fractures in the “cracked tooth syndrome” or can occur solely on root surfaces [30, 31].

## 2. Discussion

It is known that both the pulp and the periodontium are closely linked to each other, through the apical foramen, accessory canals, and dentinal tubules of the root, and one can interfere on the integrity of the other. Although there is existence of these communication routes, the mechanism of direct transmission of the periodontal infection to the pulp is still controversial. Some authors such as Rubach and Mitchell [18] affirmed that the periodontal disease may affect the pulp when there is exposure of the accessory canals through the apical foramina and the canaliculi in the furcation. Adriaens et al. [8] reported that the bacteria coming from the periodontal pockets may contaminate the pulp through the dentinal tubules that would be exposed during root planning and scaling, serving as a microorganism reservoir resulting in the recolonization of the treated root surface. Some studies [2, 38] have contradicted this idea, because even with the removal of the cementum during the periodontal therapy in vital teeth, the pulp tissue will be protected against the harmful agents through forming reparative dentin. Moreover, the dentinal fluids move towards the exterior, thereby reducing the diffusion of the harmful products of the bacteria on the exposed dentin. On the other hand, Langeland et al. [6] affirmed that only pulp would be affected by the periodontal disease if the apical foramen is involved.

The differential diagnosis of endodontic and periodontal diseases can sometimes be difficult but it is of vital importance to make a correct diagnosis so that the appropriate treatment can be provided. Endodontic-periodontal lesions present challenges to the clinician as far as diagnosis and prognosis of the involved teeth are concerned. Etiologic factors such as bacteria, fungi, and viruses as well as other various contributing factors such as trauma, root resorptions, perforations, and dental malformations also play an important role in the development and progression of such lesions.

The endo-perio lesion is a condition characterized by the association of periodontal and pulpal disease in the same dental element. This highlights the importance of taking the complete clinical history and making the right diagnosis to ensure correct prognosis and treatment. Taking into consideration all these factors and the divergences regarding the origin and direction that these infections developed, the new modified classification of these lesions has been justified.

## 3. Conclusions

Based on the current classification, it can be concluded that it is of extreme importance that the dentist should know how to differentiate between the origins of the periodontal-endodontic lesions, including all the routes of communication between the pulp and the periodontium which act as possible “bridges” for the microorganisms, thereby enabling the dissemination of the infection from one site to another.

Through this knowledge, the dentist will achieve the correct diagnosis and adequate treatment, resulting in greater chances of obtaining success in the treatment of the periodontal-endodontic lesions.

Due to the complexity of these infections, an interdisciplinary approach with a good collaboration between endodontists, Periodontist, and microbiologists is recommended.
